# Inclusion of non-medical interventions in model-based economic evaluations for tuberculosis: A scoping review

**DOI:** 10.1371/journal.pone.0290710

**Published:** 2023-08-25

**Authors:** Lauren C. Ramsay, Marina Richardson, Rafael N. Miranda, Marian Hassan, Sarah K. Brode, Elizabeth Rea, Beate Sander

**Affiliations:** 1 University of Toronto, Toronto, ON, Canada; 2 University Health Network, Toronto Health Economics and Technology Assessment Collaborative, Toronto, ON, Canada; 3 West Park Healthcare Centre, Toronto, ON, Canada; 4 Department of Medicine, University Health Network, Toronto, ON, Canada; 5 Toronto Public Health, Toronto, ON, Canada; 6 Public Health Ontario, Toronto, ON, Canada; Xiamen University - Malaysia Campus: Xiamen University - Malaysia, MALAYSIA

## Abstract

**Background:**

The economic evaluation of health interventions is important in priority setting. Several guidance documents exist to support the conduct of economic evaluations, however, there is limited guidance for the evaluation of non-medical interventions. For tuberculosis (TB), where equity-deserving groups are disproportionately impacted, assessing interventions aimed at addressing social risk factors is necessary to effectively reduce TB burden.

**Objective:**

This scoping review seeks to assess the existing literature on model-based economic evaluations of TB interventions to gauge the extent to which non-medical interventions have been evaluated in low-TB-incidence jurisdictions. As a secondary objective, this review aims to characterize key features of existing economic evaluations of medical and non-medical interventions.

**Methods:**

A literature search was conducted in the grey literature and MEDLINE, Embase, EconLit, and PsychINFO databases to September 6, 2022 following the Arksey and O’Malley framework. Eligible articles were those that used decision-analytic modeling for economic evaluation of TB interventions in low-TB-incidence jurisdictions.

**Results:**

This review identified 127 studies that met the inclusion criteria; 11 focused on prevention, 73 on detection, and 43 on treatment of TB. Only three studies (2%) evaluated non-medical interventions, including smoking reduction strategies, improving housing conditions, and providing food vouchers. All three non-medical intervention evaluations incorporated TB transmission and robust uncertainty analysis into the evaluation. The remainder of the studies evaluated direct medical interventions, eight of which were focused on specific implementation components (e.g., video observed therapy) which shared similar methodological challenges as the non-medical interventions. The majority of remaining evaluated medical interventions were focused on comparing various screening programs (e.g., immigrant screening program) and treatment regimens.

**Conclusions:**

This scoping review identified a gap in literature in the evaluation of non-medical TB interventions. However, the identified articles provided useful examples of how economic modeling can be used to explore non-traditional interventions using existing economic evaluation methods.

## Introduction

Prior to the COVID-19 pandemic, tuberculosis (TB) was responsible for the most global infectious disease deaths annually [1]. While rates of TB have generally declined in many jurisdictions (e.g., Canada, United States), to meet the World Health Organization’s End TB Strategy, these jurisdictions must continue to reduce the burden of TB [2, 3]. For example, in Canada, TB rates declined between 1940 and 1980 from over 100 cases per 100,000 population, but the decline in TB rate has remained steadier since then, with an average annual rate of approximately 5 per 100,000 population in recent years [4]. To meet the WHO goal to eliminate TB, Canada’s TB rates must still decline by 10% annually [3]. This TB rate stagnation exists even in settings with relatively robust screening, diagnostic, and treatment interventions in place. In low-incidence jurisdictions TB predominantly impacts equity-deserving groups [5], and is greatly impacted by social determinants (e.g., housing density/crowding, malnutrition [6, 7]). To effectively reduce the burden of TB in these jurisdictions, there is a need to assess interventions that target social barriers and determinants of health.

The economic evaluation of interventions is important in priority setting [8]. Model-based economic evaluations are commonly used to inform healthcare resource allocation [9, 10]. Several guidance documents exist to support the conduct of economic evaluations in health (e.g., the Canadian Agency for Drugs and Technologies in Health [CADTH], United States Panel on Cost-Effectiveness in Health and Medicine, National Institute for Health and Care Excellence), however there is limited guidance for the evaluation of non-medical interventions [8, 11, 12]. High quality models can effectively synthesize data from multiple sources, explore uncertainty, and be used as aids in decision making [9]. However, most economic evaluations focus on single biomedical interventions [10]. For TB, where social determinants of health are important intervention goals in conjunction with medical interventions, well-established methods for economic evaluations face several challenges: incorporating equity considerations, attributing intervention effects, the measurement of costs and benefits across multiple sectors, and the valuation of relevant outcomes [13]. Non-medical interventions are not necessarily planned and delivered in a healthcare setting and therefore may not be traditionally evaluated using cost-effectiveness analysis methods, despite the potential to impact TB-related health outcomes and costs [13].

Outside of economic evaluation, modeling has been used to contribute to the understanding of how socio-economic factors influence TB health outcomes including prevention and care [14–16]. One such model is S-PROTECT (Social PROTection to Enhanced Control of Tuberculosis) which uses mathematical modeling to estimate the impact of social protection interventions (e.g., cash transfers) on TB [14]. Further, a systematic review of mathematical modeling approaches used to examine the social and structural determinants of TB identified 8 articles that met their inclusion criteria published between 2008 and 2015 [15]. While it may be complex to use modeling to explore the impact of non-medical interventions on health outcomes, the examples of S-PROTECT and those identified by the systematic review demonstrate that it has the potential to contribute to policy making in an area where real-world evidence remains scarce.

This scoping review seeks to assess the existing literature on model-based economic evaluations of TB interventions to gauge the extent to which non-medical interventions have been evaluated in low-TB-incidence jurisdictions. As a secondary objective, this review aims to characterize key features of existing medical and non-medical economic evaluations.

## Methods

This study was completed following the methods outlined by the Joanna Briggs Institute Methods Manual for scoping reviews [17] based on the Arksey and O’Malley framework for Scoping Reviews [18]. Reporting followed the Preferred Reporting Items for Systematic Reviews and Meta-Analyses Scoping Review (PRISMA-ScR) extension [19]. Ethics approval was not required for this review.

### Search strategy

A literature search was conducted in MEDLINE, Embase, EconLit and PsychINFO databases from inception to September 6, 2022. A unique search strategy was developed for each database with the assistance of an information specialist with experience in systematic literature searches. The CADTH search filters for economic evaluations were used for MEDLINE and Embase [20], and these terms were translated into PsychINFO and EconLit syntax. A grey literature search was conducted on over 30 health technology assessment (HTA) and health economic agency websites following the CADTH guidelines [21]. The complete search strategy used for MEDLINE is presented in S1 Appendix.

Two reviewers screened titles and abstracts, and relevant full-text articles independently. Articles deemed relevant based on titles and abstracts were read in full to determine if they meet the eligibility criteria. Conflicts were resolved through consensus, consulting a third reviewer when necessary.

### Eligibility criteria

Eligible articles were those that used decision-analytic modeling for economic evaluation of interventions aimed at preventing, detecting, or treating TB (S2 Appendix). Studies had to report both cost and effectiveness outcomes and compare two or more interventions to be eligible for inclusion. Eligible economic evaluation types were cost-effectiveness analyses, cost-utility analyses, and cost-benefit analyses (defined in Table 1). To compare characteristics of economic evaluations in similar epidemiologic settings, eligible studies were conducted in Canada, the United States, Europe, Australia, or New Zealand due to these jurisdictions generally having lower TB incidence. While these jurisdictions have varying health systems (e.g., predominantly private system in the United States), the majority have advanced health systems with TB screening programs and treatment guidelines in place, and have a need to evaluate tailored interventions aimed at reducing the burden of TB in equity-deserving groups [5]. Review articles, comments, replies, dissertations, correspondences, conference abstracts, and editorials were excluded. Only articles that had full text available in English were included.

**Table 1:** Measurement of costs and health outcomes in economic evaluations.

Type of study	Measurement of costs	Measurement of health outcomes
Cost-effectiveness analysis	Monetary units	Natural units (e.g., life years gained, cases averted)
Cost-utility analysis	Monetary units	Healthy years (quality-adjusted life years)
Cost-benefit analysis	Monetary units	Monetary units

*Adapted from Drummond et al. (page 11) [10]

### Data charting and synthesis

Data was extracted for all articles by two authors independently. Data extracted included: publication information, study setting, type of economic evaluation, model type, intervention type, comparator strategies, outcomes measured, time horizon, discounting, and model perspective.

Extracted data was synthesized descriptively. All studies were categorized by intervention category and type to identify modeling features in different types of evaluations. Intervention groups were defined as prevention (e.g., vaccination), detection (e.g., screening programs, laboratory tests), and treatment (e.g., drug regimen, treatment adherence). Interventions were also classified as medical and non-medical. Non-medical interventions were defined as those that are not traditionally planned and/or delivered by the healthcare system but have measurable impact on health-related costs and outcomes. Conversely, medical interventions were defined as those that are directly planned and/or delivered in a healthcare setting and act on the disease and/or health outcome. Given that there is a lack of official guidance for the conduct of economic evaluations of non-medical interventions and these evaluations face specific challenges, studies that evaluated non-medical interventions were expanded upon narratively to describe the types of interventions evaluated and key methodological considerations. Within the evaluations of medical interventions, additional data was charted for studies that incorporated unique implementation considerations of a medical intervention (e.g., artificial intelligence program for observed therapy).

## Results

### Search results

**Fig 1:**
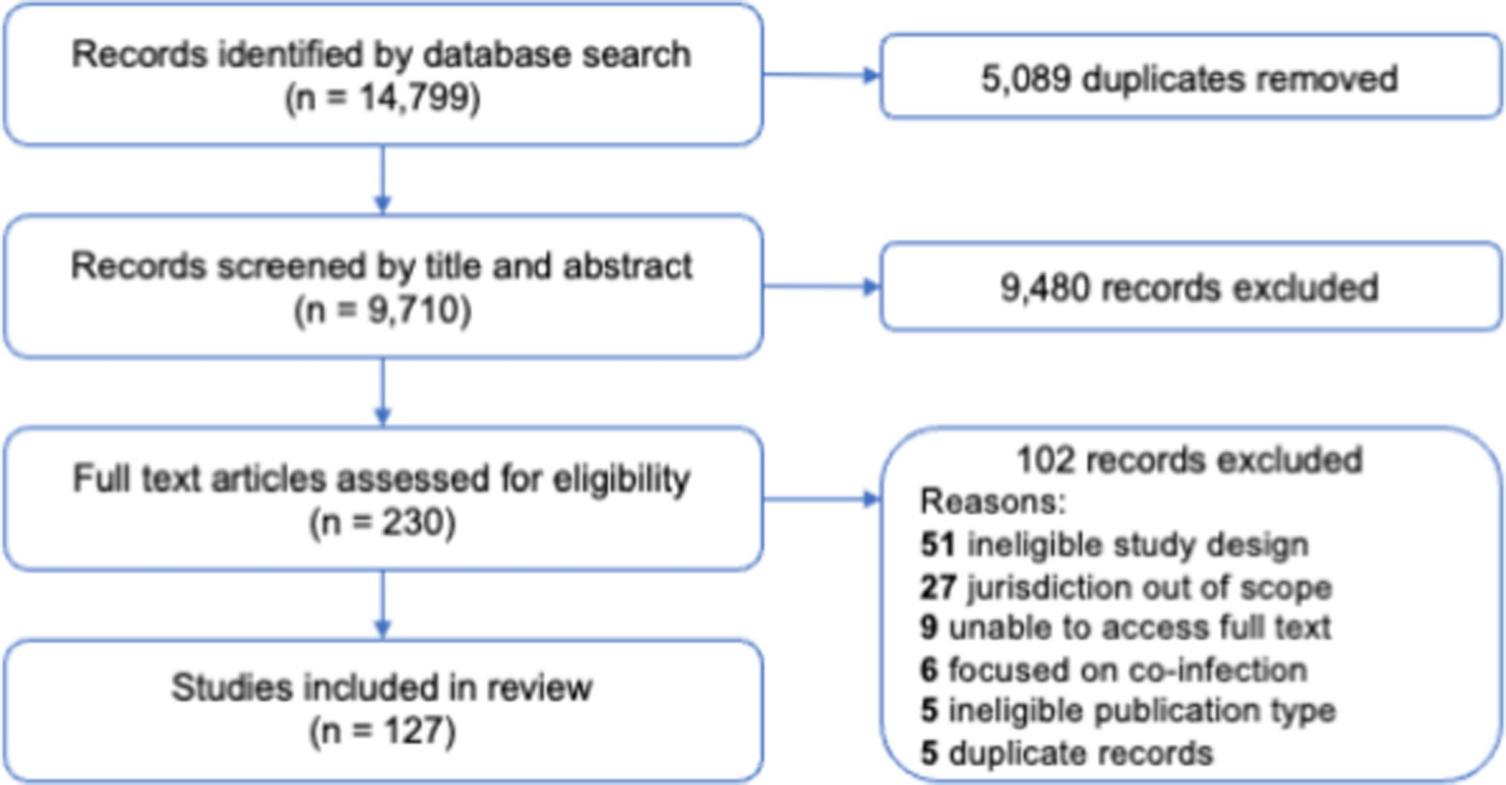
PRISMA flow chart.

The database search identified 9,710 unique articles (Fig 1). After screening titles and abstracts, 230 records were selected for full-text review. Of these, 127 studies met the inclusion criteria (S1 Table). The reasons for excluding articles at the full-text screening stage are included in Fig 1.

### Descriptive overview of included studies

**Fig 2:**
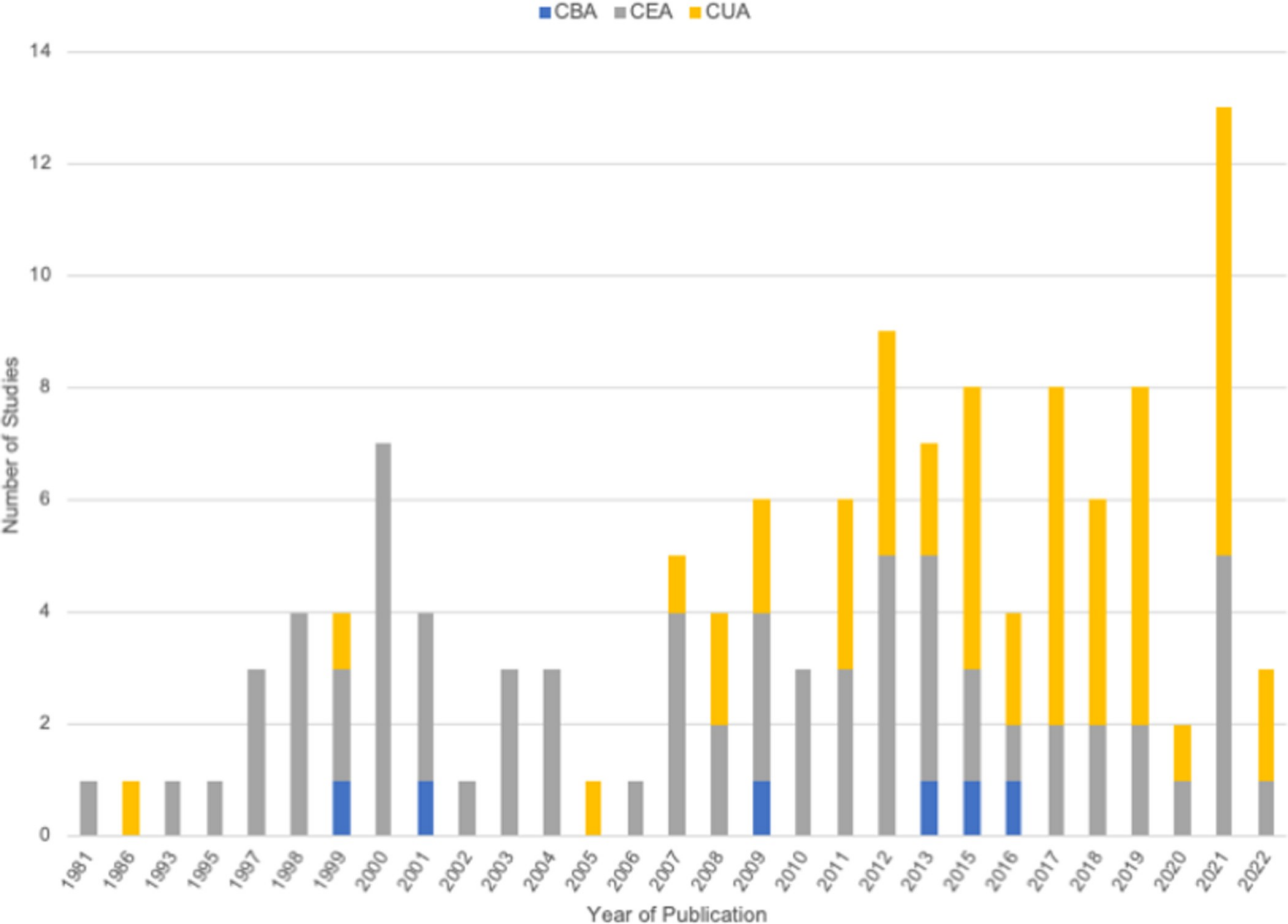
Included studies (n = 127) publication year and type of analysis. Abbreviations: CBA: cost-benefit analysis; CEA: cost-effectiveness analysis; CUA: cost-utility analysis.

Table 2 presents an overview of the included studies characteristics. Of the 127 included studies, the majority were published in the year 2010 or later (n = 77, 61%), and only 15 studies (12%) were published prior to 2000. The majority of studies were published in North America (n = 71; 56%), followed by Europe (n = 48; 38%), Australia (n = 6; 5%) and two studies that modeled multiple eligible populations. More than half of the included studies were cost-effectiveness analyses (n = 70; 55%), however, over time it appears that cost-utility analyses increased in frequency and made up a larger of proportion of analysis types (Fig 2).

**Table 2:** Summary of studies included in scoping review by intervention category.

	Prevention (n = 11)	Detection (n = 73)	Treatment (n = 43)	TOTAL (n = 127)
**Year of publication, n (% of column total)**				
Prior to 2000	3 (27)	5 (7)	7 (16)	15 (12)
2000 to 2009	4 (36)	20 (27)	11 (26)	35 (27)
2010 and later	4 (36)	48 (66)	25 (58)	77 (61)
**Region of analysis, n (% of column total)**				
North America	5 (45)	39 (54)	27 (63)	71 (56)
Europe	5 (45)	28 (38)	15 (35)	48 (38)
Australia	0	5 (7)	1 (2)	6 (5)
Other	1 (9)	1 (1)	0	2 (2)
**Intervention Type, n (% of column total)**				
Medical	9 (82)	73 (100)	42 (98)	124 (98)
Non-medical	2 (18)	0	1 (2)	3 (2)
**Type of evaluation, n (% of column total)**				
Cost-effectiveness analysis	6 (55)	40 (55)	24 (56)	70 (55)
Cost-utility analysis	3 (27)	29 (39)	19 (44)	51 (40)
Cost-benefit analysis	2 (18)	4 (5)	0	6 (5)
**Perspective of Analysis, n (% of column total)**				
Healthcare payer	8 (73)	54 (74)	28 (65)	90 (71)
Societal	2 (18)	13 (18)	12 (28)	27 (21)
Government	1 (9)	3 (4)	2 (5)	6 (5)
Other	0	3 (4)	1 (2)	4 (3)
**Model type, n (% of column total)**				
Decision Tree	2 (18)	36 (49)	17 (40)	55 (43)
Markov	3 (27)	23 (32)	22 (51)	48 (38)
Transmission	3 (27)	5 (7)	1 (2)	9 (7)
Discrete Event Simulation	0	4 (5)	0	4 (3)
Microsimulation	0	2 (3)	2 (5)	4 (3)
Other	3 (27)	3 (4)	1 (2)	7 (6)
**Time Horizon, n (% of column total)**				
Under 5 years	0	17 (23)	8 (19)	25 (20)
5–14 years	1 (9)	7 (10)	12 (28)	20 (16)
15 years to lifetime	9 (82)	43 (58)	29 (47)	72 (57)
Unclear	1 (9)	6 (8)	3 (7)	10 (8)

Included studies were categorized based on the type of intervention–medical or non-medical interventions. Only three (2%) of the identified articles evaluated non-medical interventions. The majority of identified analyses evaluated medical interventions (n = 124; 98%). Several different model types were identified; however, decision trees and Markov models were most often used (43% and 38%, respectively). The time horizon varied by study, with the majority reporting a lifetime time horizon (n = 72; 57%).

### Non-medical interventions

Three studies included a model-based economic evaluation of non-medical interventions (Table 3) [22–24]. As identifying these evaluations was the primary objective of this scoping review, the interventions evaluated, and key methodological considerations are described below.

**Table 3:** Study details for studies that evaluated non-medical interventions.

**Study & country of analysis**	**Intervention Type**	**Type of Evaluation & Time Horizon**	**Perspective**	**Model Type**	**Population**	**Comparator strategies**
N’Diaye 2019 [22] Canada	Prevention	CUA 20 years	Healthcare payer	Transmission leading to Markov model	Inuit	(1) increased tobacco taxation; (2) pharmacotherapy and counselling; (3) pharmacotherapy, counselling, and mass media; (4) the combination of all the above strategies
Doan 2019 [23] Bulgaria	Treatment	CEA 20 years	Healthcare payer	Transmission	Bulgarians	1) Short course regimen for MDR-TB 2) Scale up drug susceptibility coverage 3) Scale up food vouchers for patients under treatment 4) Transition from inpatient to ambulatory care 5) Discontinue open doors policy 6) Discontinue NGO activity
Uppal 2021 [24] Canada	Prevention	CUA 20 years	Government payer	Transmission leading to Markov model	Inuit	(1) Status quo (2) Tobacco reduction strategy (3) Heavy drinking reduction (4) Food insecurity reduction (5) Overcrowded housing reduction

Abbreviations: CEA: cost-effectiveness analysis; CUA: cost-utility analysis; MDR-TB: multi-drug resistant tuberculosis

The first study, conducted by N’Diaye and colleagues [22], assessed the potential cost-effectiveness of the impact of tobacco reduction strategies (and combinations of multiple strategies) including pharmacotherapy (a direct medical intervention) and increased tobacco taxation (a non-medical intervention) on TB-related outcomes in a Canadian Inuit population. This cost-utility analysis used a 20-year time horizon and healthcare payer perspective. This analysis included interventions planned and delivered by the healthcare system in which costs of pharmacotherapy, counseling, and media campaigns were captured in the healthcare payer perspective, and omitted costs of tobacco taxation which would be incurred only by the individual.

In the second study, Doan and colleagues [23] evaluated the health and economic outcomes of seven intervention scenarios including switching to a short course regimen for multi-drug resistant TB (MDR-TB), transitioning from inpatient to ambulatory care, and providing food vouchers for patients undergoing TB treatment (a non-medical intervention). This study was conducted from the healthcare payer perspective and predicted outcomes over a 20-year time horizon. Outcomes included TB incidence, MDR-TB incidence, TB related mortality, MDR mortality and program costs per year.

Finally, Uppal et al. considers four hypothetical programs aimed at risk factors for TB in a Canadian Inuit population: tobacco use, alcohol consumption, food insecurity, and overcrowded housing [24]. This analysis, which was conducted from a government payer perspective over a 20-year period, incorporated existing programs (e.g., inpatient camp aimed at reducing heavy drinking, construction of new housing) and scaled up the costs and health outcomes hypothetically.

All three of the evaluations of non-medical interventions incorporated TB transmission into the analysis. In the two Canadian studies, dynamic transmission modeling was used to establish TB prevalence and smoking habits in the population to inform the decision analysis using a Markov model [22, 24]. Doan and colleagues [23] used a dynamic transmission model that simulated the Bulgarian population’s risk of infection, progression, and transmission of TB over the analytic time horizon.

All three studies noted that there was a lack of evidence to inform some model input parameter values for the effectiveness of some non-medical interventions, a known challenge of economic evaluations of non-medical interventions (i.e., attribution of intervention effects) [13]. Each study mitigated the challenge of the uncertainty in efficacy data in different ways: Doan et al. transparently provided point estimates used in the model and carefully highlighted the strength of supporting evidence using the Oxford Centre for Evidence Based Medicine Levels of Evidence [23]; N’Diaye and colleagues presented deterministic and probabilistic sensitivity analyses including changing key parameters around intervention effectiveness [22]; and Uppal et al. focused on identifying thresholds of necessary program effectiveness in areas of marked uncertainty, and presented alternative scenarios including combinations of the four strategies [24]. Several health outcomes were incorporated into these analyses including quality adjusted life years (QALYs), incident TB cases, TB related deaths and TB prevalence. No included study quantitatively incorporated equity considerations into their analyses or modeled potentially relevant non-medical outcomes (e.g., housing security, educational attainment) despite modeling multi-sectoral interventions.

### Medical interventions

There were 124 economic evaluations of medical interventions identified by the literature search (S1 Table). Of these, 73 (59%) were focused on the detection of TB, 42 (34%) on treatment, and 9 (7%) on prevention. Screening programs aimed at identifying latent TB infections were frequently evaluated in low-incidence jurisdictions, as well as screening programs aimed at immigrant populations and their close contacts. Relatively few studies (n = 7) evaluated vaccination programs in low-incidence settings (where routine TB immunization is not common), and these evaluations mostly focused on vaccinating select populations (e.g., people experiencing homelessness, high risk infants). There were many different outcome measures reported across all included studies including, but not limited to, cost per QALY, cost-per-life year, cost-per-TB-case, incident TB cases, prevalent TB cases, TB-related deaths, and TB-related hospitalizations.

Among the studies that evaluated medical interventions, eight studies (7%) focused their evaluation on interventions that considered a unique implementation component (e.g., artificial intelligence platform for observed therapy as opposed to in-person directly observed therapy [DOT]) [25–32]. Details of the eight studies are included in the S2 Table. Most of the interventions identified in these studies were categorized as treatment (n = 6; 75%), and all six of these studies were focused on improving treatment adherence [25, 26, 29–32]. While evaluations of treatment adherence (including DOT) were categorized as medical interventions, these six studies evaluated more context specific implementations in their analysis and thus were of particular interest from a modeling perspective. The implementation components that were identified to improve treatment adherence included video-observed therapy, financial incentives, contingency contracts for adolescents with their parents, and an artificial intelligence monitoring platform. The remaining two studies evaluated a novel contact tracing approach [27] and a mobile find and treat service for hard-to-reach populations [28]. Five of the eight studies were conducted alongside a study that assessed the effectiveness of the intervention which helped to inform the economic model [25, 26, 28, 29, 31]. While these studies were evaluating medical interventions, they were summarized in more detail because of potential similarities in modeling considerations as non-medical interventions such as navigating uncertainty and generalizability of the model outcomes.

## Discussion

This scoping review provided a narrative summary of model-based economic evaluations of non-medical interventions in low TB-incidence countries. Additionally, it presented evaluation features of the 127 identified model-based economic evaluations on TB interventions in high-resource settings (e.g., model type, time horizon, type of analysis). The majority of studies evaluated only direct medical interventions. Only three studies that evaluated non-medical interventions were identified in this review.

The lack of high-quality data to inform economic models poses challenges for both non-medical interventions and context-specific analyses of medical interventions. The three non-medical intervention evaluations addressed this uncertainty through transparency in reporting and sensitivity/scenario analyses, thus providing examples of how economic modeling can be informative in the absence of clinical trials or robust observational data to inform short term and long-term extrapolation of clinical efficacy parameters. Of the eight studies that evaluated a unique implementations of a medical intervention, five of them were conducted alongside observational or experimental research studies which informed key model parameters thus overcoming data collection concerns. The non-medical intervention evaluations, along with the studies focused on implementation components demonstrate that economic evaluation methods can be used successfully even in the presence of uncertainty. In fact, the use of decision-analytic modeling in areas where other evidence remains scarce or difficult to collect may be a useful tool for decision makers.

The perspective of analysis may influence cost-effectiveness conclusions regardless of the type of intervention studied; however, evaluations focused on interventions that may be planned or implemented outside the healthcare system exemplify this well. For example, in one of the identified non-medical intervention evaluations, the authors included the costs associated with improving housing conditions using a government payer perspective that would have been omitted using a healthcare payer perspective [24]. A societal perspective would further capture relevant costs to individuals such as the increased cost of tobacco products following increased tobacco taxation included in N’Diaye et al.’s evaluation [22]. In this scoping review, the majority of studies were conducted from a healthcare payer perspective. This may be because jurisdictional economic evaluation guidelines recommend the healthcare payer perspective [8, 11]. Conducting analyses under a single analytic perspective makes it difficult to incorporate meaningful societal impacts and equity considerations due to the narrow measurement of costs and health outcomes [33]. This may impede public funding of interventions or lead to unintended consequences such as the shifting of costs towards patients and their families. For these reasons, the movement towards broader perspectives, such as a government payer and societal should be considered, especially for the evaluation of interventions with costs and outcomes falling in different sectors [34].

Prior literature on the economic evaluation of complex interventions (i.e., interventions that are made up of several components that may interact with one another) note that while more resource intensive (i.e., researcher time, funding) it is still possible to conduct high quality economic evaluations, though may require deviations or additions to traditional evaluation methods [35, 36]. Specifically related to TB, modeling studies, including economic evaluations, can help policy makers identify effective interventions and populations to prioritize; however, consideration must be given to uncertainty in key inputs (e.g., effectiveness, costs of interventions) [37].

As with all research, this study has some limitations. The eligibility criteria was limited to publications with full texts available in English which could introduce language bias and lead to the underrepresentation of European studies that would have otherwise been included. However, a random subset (10%) of non-English language articles were reviewed and none met the remaining eligibility criteria, and the majority were published in languages native to ineligible jurisdictions (e.g., China). There were also nine studies that the authors were unable to access the full texts for and were thus excluded.

This scoping review aimed to identify only economic evaluations, and thus excluded other modeling studies that did not include an economic component. However, a prior systematic review identified modeling studies that considered social and structural determinants of health related to TB which provided useful examples of how modeling can be used to examine these questions [15]. Future collaboration between groups who conduct economic evaluations and epidemiological models would be warranted to improve the economic evaluation methods around non-medical and context-specific interventions. Further, in the study selection process, articles that focused on populations outside of North America, Europe and Australia were excluded to maintain a level of comparability across epidemiologically similar jurisdictions. Given that TB is highly endemic outside of these regions, this study would have excluded economic evaluations in those settings. It is important to emphasize that the decision to limit the scope of this review was not arbitrary but based on feasibility and comparability considerations. Due to limited resources and the nature of the research question, it was necessary to focus the review on a specific subset of countries. Nevertheless, the economic evaluation of non-medical interventions in high-TB-incidence countries remains an important area of research, and one that may provide valuable examples and insights into conducting these evaluations in the future. To our knowledge, a scoping review of this nature has yet to be conducted in alternative jurisdictions and this presents an important area for future research.

## Conclusions

This scoping review identified a gap in literature in the evaluation of non-medical TB interventions in the included jurisdictions. However, the identified articles provided useful examples of how economic modeling can be used to explore non-medical and context-specific interventions using existing economic evaluation methods. These evaluations have the potential to assess alternative outcomes such as financial security, improved capabilities, and changes in societal costs that may be associated with non-medical interventions. An update of this review in the future would help to establish if there is a trend towards the economic evaluation of non-medical interventions and if future evaluations estimate effects on non-medical outcomes.

## Supporting information

S1 Appendix Sample search strategy for MEDLINE. (DOCX)

S2 Appendix Definition of model-based economic evaluation applied in this study. (DOCX)

S1 Table Table of included studies (n = 127). (DOCX)

S2 Table Table with study details of evaluations that focused on unique implementation components of a health intervention (n = 8). (DOCX)

S1 Checklist Preferred Reporting Items for Systematic reviews and Meta-Analyses extension for Scoping Reviews (PRISMA-ScR) checklist. (PDF)
