# Supplementary material for: Inclusion of non-medical interventions in model-based economic evaluations for tuberculosis: A scoping review
Source: PLoS One. 2023 Aug 25;18(8):e0290710. doi: 10.1371/journal.pone.0290710 (PMC10456154; doi:10.1371/journal.pone.0290710)
Supplement: S1 Appendix — (DOCX) [file pone.0290710.s001.docx]

**APPENDIX A**

Sample search strategy for MEDLINE

Reference: Strings attached: CADTH database search filters [Internet]. Ottawa: CADTH; 2016. [cited 2019 10 09]. Available from: [/resources/finding-evidence](https://www.cadth.ca/resources/finding-evidence)

Database: Ovid MEDLINE: Epub Ahead of Print, In-Process & Other Non-Indexed Citations, Ovid MEDLINE® Daily and Ovid MEDLINE® <1946-Present>

Search Strategy:

--------------------------------------------------------------------------------

1 Economics/

2 exp "Costs and Cost Analysis"/

3 Economics, Nursing/

4 Economics, Medical/

5 Economics, Pharmaceutical/

6 exp Economics, Hospital/

7 Economics, Dental/

8 exp "Fees and Charges"/

9 exp Budgets/

10 budget*.ti,ab,kf.

11 (economic* or cost or costs or costly or costing or price or prices or pricing or pharmacoeconomic* or pharmaco-economic* or expenditure or expenditures or expense or expenses or financial or finance or finances or financed).ti,kf. (218117)

12 (economic* or cost or costs or costly or costing or price or prices or pricing or pharmacoeconomic* or pharmaco-economic* or expenditure or expenditures or expense or expenses or financial or finance or finances or financed).ab. /freq=2

13 (cost* adj2 (effective* or utilit* or benefit* or minimi* or analy* or outcome or outcomes)).ab,kf.

14 (value adj2 (money or monetary)).ti,ab,kf.

15 exp models, economic/

16 economic model*.ab,kf.

17 markov chains/

18 markov.ti,ab,kf.

19 monte carlo method/

20 monte carlo.ti,ab,kf.

21 exp Decision Theory/

22 (decision* adj2 (tree* or analy* or model*)).ti,ab,kf.

23 or/1-22

24 exp Tuberculosis, Multidrug-Resistant/ or exp Tuberculosis/ or exp Mycobacterium tuberculosis/ or exp Tuberculosis, Pulmonary/ or exp Latent Tuberculosis/ or exp Tuberculosis Vaccines/ or exp Extensively Drug-Resistant Tuberculosis/ or tuberculosis.mp.

25 23 and 24

26 limit 25 to humans

27 (letter or Editorial or historical article).pt.

28 26 not 27
