# Supplementary material for: Inclusion of non-medical interventions in model-based economic evaluations for tuberculosis: A scoping review
Source: PLoS One. 2023 Aug 25;18(8):e0290710. doi: 10.1371/journal.pone.0290710 (PMC10456154; doi:10.1371/journal.pone.0290710)
Supplement: S2 Appendix — (DOCX) [file pone.0290710.s002.docx]

**Appendix B**

Definition of model-based economic evaluation applied in this study

We referred to the literature and discussed in depth the defining features of a model-based economic evaluation. Some of these features included that they relied on data, often from multiple sources, to simulate and quantify the impact of different interventions or policies on economic and health outcomes. We also established that to be considered a model-based economic evaluation, studies should have the ability to incorporate uncertainty (i.e., have the ability to assess parameter uncertainty through sensitivity or scenario analyses), consider a relevant time horizon (e.g., consider a long term perspective based on the available data), compare two or more alternatives, and allow for an incremental analysis. It is important to note that these features represent common characteristics of model-based economic evaluations, but specific methodologies and approaches may vary depending on the research question, context, and available data.
