# Supplementary material for: Inclusion of non-medical interventions in model-based economic evaluations for tuberculosis: A scoping review
Source: PLoS One. 2023 Aug 25;18(8):e0290710. doi: 10.1371/journal.pone.0290710 (PMC10456154; doi:10.1371/journal.pone.0290710)
Supplement: S1 Table — (DOCX) [file pone.0290710.s003.docx]

**Table S1.** Table of included studies in review (n = 127)

| **Study** | **Title** | **Journal** | **Region** | **Intervention**  **Category** | **Intervention**  **Type** | **Evaluation**  **Type** |
| --- | --- | --- | --- | --- | --- | --- |
| Barrett-Connor 1981 | Nosocomial tuberculosis in physicians: an analysis of direct costs of prevention and disease. | Preventive medicine | NA | Prevention | Direct | CEA |
| Snider 1986 | Preventive therapy with isoniazid. Cost-effectiveness of different durations of therapy. | JAMA | NA | Treatment | Direct | CUA |
| Nettleman 1993 | Use of BCG vaccine in shelters for the homeless. A decision analysis. | Chest | NA | Prevention | Direct | CEA |
| Mohle-Boetani 1995 | School-based screening for tuberculous infection. A cost-benefit analysis. | JAMA | NA | Detection | Direct | CEA |
| Salpeter 1997 | Monitored isoniazid prophylaxis for low-risk tuberculin reactors older than 35 years of age: a risk-benefit and cost-effectiveness analysis. | Annals of internal medicine | NA | Treatment | Direct | CEA |
| Nettleman 1997 | The cost-effectiveness of preventing tuberculosis in physicians using tuberculin skin testing or a hypothetical vaccine. | Archives of internal medicine | NA | Detection | Direct | CEA |
| Burman 1997 | A cost-effectiveness analysis of directly observed therapy vs self-administered therapy for treatment of tuberculosis. | Chest | NA | Treatment | Direct | CEA |
| Nicas 1998 | A risk/cost analysis of alternative screening intervals for occupational tuberculosis infection. | American Industrial Hygiene Association journal | NA | Detection | Direct | CEA |
| Brewer 1998 | An effectiveness and cost analysis of presumptive treatment for Mycobacterium tuberculosis. | American journal of infection control | NA | Treatment | Direct | CEA |
| Migliori 1998 | Cost-effectiveness analysis of tuberculosis control policies in Ivanovo Oblast, Russian Federation. Ivanovo Tuberculosis Project Study Group. | Bulletin of the World Health Organization | Europe | Treatment | Direct | CEA |
| Palmer 1998 | A model of the cost-effectiveness of directly observed therapy for treatment of tuberculosis. | Journal of public health management and practice | NA | Treatment | Direct | CEA |
| Snyder 1999 | Tuberculosis prevention in methadone maintenance clinics. Effectiveness and cost-effectiveness. | American journal of respiratory and critical care medicine | NA | Detection | Direct | CEA |
| Snyder 1999 | Cost-effectiveness analysis of directly observed therapy for patients with tuberculosis at low risk for treatment default. | American journal of respiratory and critical care medicine | NA | Treatment | Direct | CEA |
| Pathania 1999 | A cost-benefit analysis of BCG revaccination in the Czech Republic. | Vaccine | Europe | Prevention | Direct | CBA |
| Marchand 1999 | Cost-effectiveness of screening compared to case-finding approaches to tuberculosis in long-term care facilities for the elderly. | International journal of epidemiology | NA | Detection | Direct | CUA |
| Jasmer 2000 | Twelve months of isoniazid compared with four months of isoniazid and rifampin for persons with radiographic evidence of previous tuberculosis: an outcome and cost-effectiveness analysis. | American journal of respiratory and critical care medicine | NA | Treatment | Direct | CEA |
| Boggess 2000 | Antepartum or postpartum isoniazid treatment of latent tuberculosis infection. | Obstetrics and gynecology | NA | Treatment | Direct | CEA |
| Schwartzman 2000 | Tuberculosis screening of immigrants to low-prevalence countries. A cost-effectiveness analysis. | American journal of respiratory and critical care medicine | NA | Detection | Direct | CEA |
| Chaulk 2000 | Modeling the epidemiology and economics of directly observed therapy in Baltimore. | The international journal of tuberculosis and lung disease | NA | Treatment | Direct | CEA |
| Macintyre 2000 | The cost-effectiveness of evidence-based guidelines and practice for screening and prevention of tuberculosis. | Health economics | Aus | Detection | Direct | CEA |
| Lowin 2000 | Cost effectiveness analysis of school based Mantoux screening for TB infection. | Australian and New Zealand journal of public health | Aus | Detection | Direct | CEA |
| Dasgupta 2000 | Comparison of cost-effectiveness of tuberculosis screening of close contacts and foreign-born populations. | American journal of respiratory and critical care medicine | NA | Detection | Direct | CEA |
| Perlman 2001 | Cost-effectiveness of tuberculosis screening and observed preventive therapy for active drug injectors at a syringe-exchange program. | Journal of urban health : bulletin of the New York Academy of Medicine | NA | Treatment | Implementation | CEA |
| Jones 2001 | Miniature chest radiograph screening for tuberculosis in jails: a cost-effectiveness analysis. | American journal of respiratory and critical care medicine | NA | Detection | Direct | CEA |
| Bishai 2001 | Modeling the economic benefits of better TB vaccines. | The international journal of tuberculosis and lung disease | Other | Prevention | Direct | CBA |
| Wilton 2001 | Directly observed treatment for multidrug-resistant tuberculosis: an economic evaluation in the United States of America and South Africa. | The international journal of tuberculosis and lung disease | NA | Treatment | Direct | CEA |
| Khan 2002 | Global drug-resistance patterns and the management of latent tuberculosis infection in immigrants to the United States. | The New England journal of medicine | NA | Detection | Direct | CEA |
| Hersh 2003 | Dual skin testing for latent tuberculosis infection: a decision analysis. | American journal of preventive medicine | NA | Detection | Direct | CEA |
| Hersh 2003 | A cost-effectiveness analysis of universal versus selective immunization with Mycobacterium bovis bacille Calmette-Guerin in Finland. | The international journal of tuberculosis and lung disease | Europe | Prevention | Direct | CEA |
| Dowdy 2003 | Cost-effectiveness analysis of the gen-probe amplified mycobacterium tuberculosis direct test as used routinely on smear-positive respiratory specimens. | Journal of clinical microbiology | NA | Detection | Direct | CEA |
| Salpeter 2004 | Screening and treatment of latent tuberculosis among healthcare workers at low, moderate, and high risk for tuberculosis exposure: a cost-effectiveness analysis. | Infection control and hospital epidemiology | NA | Detection | Direct | CEA |
| Jasmer 2004 | Short-course rifampin and pyrazinamide compared with isoniazid for latent tuberculosis infection: a cost-effectiveness analysis based on a multicenter clinical trial. | Clinical infectious diseases | NA | Treatment | Direct | CEA |
| Rajalahti 2004 | Economic evaluation of the use of PCR assay in diagnosing pulmonary TB in a low-incidence area. | The European respiratory journal | Europe | Detection | Direct | CEA |
| Diel 2005 | Cost-effectiveness of isoniazid chemoprevention in close contacts. | The European respiratory journal | Europe | Treatment | Direct | CUA |
| Porco 2006 | Cost-effectiveness of tuberculosis evaluation and treatment of newly-arrived immigrants. | BMC public health | NA | Detection | Direct | CEA |
| Kominski 2007 | Costs and cost-effectiveness of adolescent compliance with treatment for latent tuberculosis infection: results from a randomized trial. | The Journal of adolescent health | NA | Treatment | Implementation | CUA |
| Oxlade 2007 | Interferon-gamma release assays and TB screening in high-income countries: a cost-effectiveness analysis. | The international journal of tuberculosis and lung disease | NA | Detection | Direct | CEA |
| Flaherman 2007 | Cost-effectiveness of alternative strategies for tuberculosis screening before kindergarten entry. | Pediatrics | NA | Detection | Direct | CEA |
| Diel 2007 | Cost-effectiveness of interferon-gamma release assay screening for latent tuberculosis infection treatment in Germany. | Chest | Europe | Detection | Direct | CEA |
| Diel 2007 | Cost-effectiveness of interferon-gamma release assay testing for the treatment of latent tuberculosis. | The European respiratory journal | Europe | Detection | Direct | CEA |
| Tan 2008 | Cost-effectiveness of LTBI treatment for TB contacts in British Columbia. | Value in health | NA | Treatment | Direct | CUA |
| Marra 2008 | Cost-effectiveness of a new interferon-based blood assay, QuantiFERON-TB Gold, in screening tuberculosis contacts. | The international journal of tuberculosis and lung disease | NA | Detection | Direct | CUA |
| Tan 2008 | Tuberculosis screening of travelers to higher-incidence countries: a cost-effectiveness analysis. | BMC public health | NA | Detection | Direct | CEA |
| Guerra 2008 | Cost-effectiveness of different strategies for amplified Mycobacterium tuberculosis direct testing for cases of pulmonary tuberculosis. | Journal of clinical microbiology | NA | Detection | Direct | CEA |
| Diel 2009 | Enhanced cost-benefit analysis of strategies for LTBI screening and INH chemoprevention in Germany. | Respiratory medicine | Europe | Detection | Direct | CBA |
| dePerio 2009 | Cost-effectiveness of interferon gamma release assays vs tuberculin skin tests in health care workers. | Archives of internal medicine | NA | Detection | Direct | CUA |
| Finnell 2009 | Latent tuberculosis infection in children: a call for revised treatment guidelines. | Pediatrics | NA | Treatment | Direct | CEA |
| Holland 2009 | Costs and cost-effectiveness of four treatment regimens for latent tuberculosis infection. | American journal of respiratory and critical care medicine | NA | Treatment | Direct | CUA |
| Pisu 2009 | Targeted tuberculosis contact investigation saves money without sacrificing health. | Journal of public health management and practice | NA | Prevention | Implementation | CEA |
| Altes 2009 | Targeted BCG vaccination against severe tuberculosis in low-prevalence settings: epidemiologic and economic assessment. | Epidemiology | Europe | Prevention | Direct | CEA |
| Pooran 2010 | Different screening strategies (single or dual) for the diagnosis of suspected latent tuberculosis: a cost effectiveness analysis. | BMC pulmonary medicine | Europe | Detection | Direct | CEA |
| Deuffic-Burban 2010 | Cost-effectiveness of QuantiFERON-TB test vs. tuberculin skin test in the diagnosis of latent tuberculosis infection. | The international journal of tuberculosis and lung disease | Europe | Detection | Direct | CEA |
| Aspler 2010 | Impact of treatment completion, intolerance and adverse events on health system costs in a randomised trial of 4 months rifampin or 9 months isoniazid for latent TB. | Thorax | NA | Treatment | Direct | CEA |
| Pareek 2011 | Screening of immigrants in the UK for imported latent tuberculosis: a multicentre cohort study and cost-effectiveness analysis. | The Lancet. Infectious diseases | Europe | Detection | Direct | CEA |
| Linas 2011 | Priorities for screening and treatment of latent tuberculosis infection in the United States. | American journal of respiratory and critical care medicine | NA | Detection | Direct | CUA |
| Holland 2011 | Potential economic viability of two proposed rifapentine-based regimens for treatment of latent tuberculosis infection. | PloS one | NA | Treatment | Direct | CUA |
| Mancuso 2011 | Cost-effectiveness analysis of targeted and sequential screening strategies for latent tuberculosis. | The international journal of tuberculosis and lung disease | NA | Detection | Direct | CEA |
| Esfahani 2011 | Potential cost-effectiveness of rifampin vs. isoniazid for latent tuberculosis: implications for future clinical trials. | The international journal of tuberculosis and lung disease | NA | Treatment | Direct | CEA |
| Jit 2011 | Dedicated outreach service for hard to reach patients with tuberculosis in London: observational study and economic evaluation. | BMJ (Clinical research ed.) | Europe | Detection | Implementation | CUA |
| delCampo 2012 | Cost-effectiveness of different screening strategies (single or dual) for the diagnosis of tuberculosis infection in healthcare workers. | Infection control and hospital epidemiology | Europe | Detection | Direct | CEA |
| Shah 2012 | QuantiFERON-TB gold in-tube implementation for latent tuberculosis diagnosis in a public health clinic: a cost-effectiveness analysis. | BMC infectious diseases | NA | Detection | Direct | CUA |
| Holland 2012 | Strategies for treating latent multiple-drug resistant tuberculosis: a decision analysis. | PloS one | NA | Treatment | Direct | CUA |
| Pina 2012 | Is isoniazid for 6 months more cost-effective than isoniazid for 9 months?. | The international journal of tuberculosis and lung disease | Europe | Treatment | Direct | CEA |
| Winetsky 2012 | Screening and rapid molecular diagnosis of tuberculosis in prisons in Russia and Eastern Europe: a cost-effectiveness analysis. | PLoS medicine | Europe | Detection | Direct | CUA |
| Wade 2012 | Home videophones improve direct observation in tuberculosis treatment: a mixed methods evaluation. | PloS one | Aus | Treatment | Implementation | CEA |
| Hughes 2012 | The cost effectiveness of Nucleic Acid Amplification Techniques for the diagnosis of tuberculosis. | Respiratory medicine | Europe | Detection | Direct | CUA |
| Floyd 2012 | Cost and cost-effectiveness of multidrug-resistant tuberculosis treatment in Estonia and Russia. | The European respiratory journal | Europe | Treatment | Direct | CEA |
| Eralp 2012 | Screening of healthcare workers for tuberculosis: Development and validation of a new health economic model to inform practice | BMJ Open | Europe | Detection | Direct | CEA |
| Pareek 2013 | Community-based evaluation of immigrant tuberculosis screening using interferon gamma release assays and tuberculin skin testing: observational study and economic analysis. | Thorax | Europe | Detection | Direct | CEA |
| Pina 2013 | Cost-effectiveness of rifampin for 4 months and isoniazid for 9 months in the treatment of tuberculosis infection. | European journal of clinical microbiology & infectious diseases | Europe | Treatment | Direct | CEA |
| Pina 2013 | Cost-effectiveness of rifampin for 4 months and isoniazid for 6 months in the treatment of tuberculosis infection. | Respiratory medicine | Europe | Treatment | Direct | CEA |
| Shepardson 2013 | Cost-effectiveness of a 12-dose regimen for treating latent tuberculous infection in the United States. | The international journal of tuberculosis and lung disease | NA | Treatment | Direct | CUA |
| Verma 2013 | Tuberculosis screening for long-term care: a cost-effectiveness analysis. | The international journal of tuberculosis and lung disease | NA | Detection | Direct | CEA |
| Choi 2013 | Cost-effectiveness of Xpert MTB/RIF for diagnosing pulmonary tuberculosis in the United States. | The international journal of tuberculosis and lung disease | NA | Detection | Direct | CUA |
| Millman 2013 | Rapid molecular testing for TB to guide respiratory isolation in the U.S.: a cost-benefit analysis. | PloS one | NA | Detection | Direct | CBA |
| Wingate 2015 | Cost-Effectiveness of Screening and Treating Foreign-Born Students for Tuberculosis before Entering the United States. | PloS one | NA | Detection | Direct | CEA |
| Wingate 2015 | A cost-benefit analysis of a proposed overseas refugee latent tuberculosis infection screening and treatment program. | BMC public health | NA | Detection | Direct | CBA |
| Fox 2015 | Fluoroquinolone therapy for the prevention of multidrug-desistant tuberculosis in contacts: A cost-effectiveness analysis | American Journal of Respiratory and Critical Care Medicine | NA | Treatment | Direct | CUA |
| Nguyen 2015 | Strengthening tuberculosis control overseas: who benefits?. | Value in health | Aus | Detection | Direct | CUA |
| Diel 2015 | Cost effectiveness of treating multi-drug resistant tuberculosis by adding DeltybaTM to background regimens in Germany. | Respiratory medicine | Europe | Treatment | Direct | CUA |
| Drobniewski 2015 | Systematic review, meta-analysis and economic modelling of molecular diagnostic tests for antibiotic resistance in tuberculosis. | Health technology assessment | Europe | Detection | Direct | CUA |
| Wolfson 2015 | Cost-effectiveness of adding bedaquiline to drug regimens for the treatment of multidrug-resistant tuberculosis in the UK. | PloS one | Europe | Treatment | Direct | CUA |
| Jakab 2015 | Consolidated Action Plan to Prevent and Combat Multidrug- and Extensively Drug-resistant Tuberculosis in the WHO European Region 2011-2015: Cost-effectiveness analysis | Tuberculosis | Europe | Prevention | Direct | CEA |
| Auguste 2016 | Accurate diagnosis of latent tuberculosis in children, people who are immunocompromised or at risk from immunosuppression and recent arrivals from countries with a high incidence of tuberculosis: systematic review and economic evaluation. | Health technology assessment | Europe | Detection | Direct | CUA |
| Mears 2016 | The prospective evaluation of the TB strain typing service in England: a mixed methods study. | Thorax | Europe | Detection | Direct | CUA |
| Diel 2016 | Cost-benefit analysis of Xpert MTB/RIF for tuberculosis suspects in German hospitals. | The European respiratory journal | Europe | Detection | Direct | CBA |
| Oxlade 2016 | XpertMTB/RIF for the Diagnosis of Tuberculosis in a Remote Arctic Setting: Impact on Cost and Time to Treatment Initiation. | PloS one | NA | Detection | Direct | CEA |
| Campbell 2017 | Cost-effectiveness of post-landing latent tuberculosis infection control strategies in new migrants to Canada. | PloS one | NA | Detection | Direct | CUA |
| Tasillo 2017 | Cost-effectiveness of Testing and Treatment for Latent Tuberculosis Infection in Residents Born Outside the United States With and Without Medical Comorbidities in a Simulation Model. | JAMA internal medicine | NA | Detection | Direct | CUA |
| Patel 2017 | Burden of non-adherence to latent tuberculosis infection drug therapy and the potential cost-effectiveness of adherence interventions in Canada: a simulation study. | BMJ open | NA | Treatment | Implementation | CUA |
| Haukaas 2017 | Immigrant screening for latent tuberculosis in Norway: a cost-effectiveness analysis. | The European journal of health economics | Europe | Detection | Direct | CEA |
| Mullie 2017 | Revisiting annual screening for latent tuberculosis infection in healthcare workers: a cost-effectiveness analysis. | BMC medicine | NA | Detection | Direct | CUA |
| Herraez 2017 | Cost-effectiveness study of the microbiological diagnosis of tuberculosis using geneXpert MTB/RIF. | Enfermedades infecciosas y microbiologia clinica | Europe | Detection | Direct | CUA |
| Cowan 2017 | Clinical Impact and Cost-effectiveness of Xpert MTB/RIF Testing in Hospitalized Patients With Presumptive Pulmonary Tuberculosis in the United States. | Clinical infectious diseases | NA | Detection | Direct | CEA |
| Wirth 2017 | Cost-effectiveness of adding novel or group 5 interventions to a background regimen for the treatment of multidrug-resistant tuberculosis in Germany. | BMC health services research | Europe | Treatment | Direct | CUA |
| Ionescu 2018 | Bedaquiline- versus injectable-containing drug-resistant tuberculosis regimens: a cost-effectiveness analysis. | Expert review of pharmacoeconomics & outcomes research | Europe | Treatment | Direct | CEA |
| Abubakar 2018 | Two interferon gamma release assays for predicting active tuberculosis: the UK PREDICT TB prognostic test study. | Health technology assessment | Europe | Detection | Direct | CUA |
| Teljeur 2018 | Economic Evaluation of Selective Neonatal Bacillus Calmette-Guerin Vaccination of High-risk Infants in Ireland. | The Pediatric infectious disease journal | Europe | Prevention | Direct | CUA |
| Maskery 2018 | Economic analysis of CDC's culture- and smear-based tuberculosis instructions for Filipino immigrants. | The international journal of tuberculosis and lung disease | NA | Detection | Direct | CEA |
| Zuur 2018 | Cost-utility analysis of high-dose treatment for intermediate-susceptible, dose-dependent tuberculosis patients. | The international journal of tuberculosis and lung disease | Europe | Treatment | Direct | CUA |
| Plavinskiy 2018 | Economic effectiveness of interferon gamma in the treatment of tuberculosis | Research Journal of Pharmaceutical, Biological and Chemical Sciences | Europe | Treatment | Direct | CUA |
| Usemann 2019 | Cost-effectiveness of tuberculosis screening for migrant children in a low-incidence country | International Journal of Tuberculosis and Lung Disease | Europe | Detection | Direct | CEA |
| Campbell 2019 | Cost-effectiveness of latent tuberculosis infection screening before immigration to low-incidence countries | Emerging Infectious Diseases | NA | Detection | Direct | CUA |
| Doan 2019 | Cost-effectiveness of 3 months of weekly rifapentine and isoniazid compared with other standard treatment regimens for latent tuberculosis infection: A decision analysis study | Journal of Antimicrobial Chemotherapy | NA | Treatment | Direct | CUA |
| Goodell 2019 | Outlook for tuberculosis elimination in California: An individual-based stochastic model | PLoS ONE | NA | Detection | Direct | CUA |
| Cavany 2019 | Should NICE reconsider the 2016 UK guidelines on TB contact tracing? A cost-effectiveness analysis of contact investigations in London. | Thorax | Europe | Detection | Direct | CUA |
| N'Diaye 2019 | The potential impact and cost-effectiveness of tobacco reduction strategies for tuberculosis prevention in Canadian Inuit communities. | BMC medicine | NA | Prevention | Non-health | CUA |
| Takwoingi 2019 | Interferon gamma release assays for diagnostic evaluation of active tuberculosis (IDEA): Test accuracy study and economic evaluation | Health Technology Assessment | Europe | Detection | Direct | CUA |
| Doan 2019 | Strategic investment in tuberculosis control in the Republic of Bulgaria | Epidemiology and Infection | Europe | Treatment | Non-health | CEA |
| Jo 2020 | Model-based Cost-effectiveness of State-level Latent  Tuberculosis Interventions in California, Florida, New York, and Texas | Clinical infectious diseases | NA | Detection | Direct | CUA |
| Wahedi 2020 | Cost-effectiveness of targeted screening for active pulmonary tuberculosis among asylum- seekers: A modelling study with screening data from a German federal state (2002-2015) | PloS one | Europe | Detection | Direct | CEA |
| Shedrawy 2021 | Cost-effectiveness of the latent tuberculosis screening program for migrants in Stockholm Region | European Journal of Health Economics | Europe | Detection | Direct | CUA |
| Pease 2021 | Cost-effectiveness analysis of 3 months of weekly rifapentine and isoniazid compared to isoniazid monotherapy in a Canadian arctic setting | BMJ open | NA | Treatment | Direct | CUA |
| Ilaiwy 2021 | Cost effectiveness of three months of rifapentine and isoniazid for latent tuberculosis in Syrian refugees | Journal of Clinical Tuberculosis and Other Mycobaterial Diseases | Europe | Treatment | Direct | CUA |
| Dale 2021 | Modeling the Cost-Effectiveness of Latent Tuberculosis Screening and Treatment Strategies in Recent Migrants to a Low-Incidence Setting | American Journal of Epidemiology | Aus | Detection | Direct | CUA |
| Uppal 2021 | Social and behavioral risk reduction strategies for tuberculosis prevention in Canadian Inuit communities: a cost- effectiveness analysis | BMC public health | NA | Prevention | Non-health | CUA |
| Salcedo 2021 | Cost-effectiveness of artificial intelligence monitoring for active tuberculosis treatment: A modeling study | PloS one | NA | Treatment | Implementation | CUA |
| Hickey 2021 | Evaluation of screening strategies for pulmonary tuberculosis among hospitalized patients in a low-burden setting: cost-effectiveness of GeneXpert MTB/RIF compared to smear microscopy | Infection control and hospital epidemiology | NA | Detection | Direct | CEA |
| Gosce 2021 | Tackling TB in migrants arriving at Europe’s southern border | International Journal of Infectious Diseases | Europe | Detection | Direct | CEA |
| Fekadu 2021 | Cost-effectiveness of video-observed therapy for ambulatory management of active tuberculosis during the COVID-19 pandemic in a high-income country | International Journal of Infectious Diseases | NA | Treatment | Implementation | CEA |
| Mugwagwa 2021 | Using molecular testing and whole-genome sequencing for tuberculosis diagnosis in a low-burden setting: a cost-effectiveness analysis using transmission-dynamic modelling | BMJ Journals - Thorax | Europe | Detection | Direct | CUA |
| Uppal 2021 | Active screening for tuberculosis in high-incidence Inuit communities in Canada: a cost-effectiveness analysis. | CMAJ | NA | Detection | Direct | CEA |
| Marx 2021 | Targeting screening and treatment for latent tuberculosis infection towards asylum seekers from high-incidence countries - a model-based cost-effectiveness analysis. | BMC public health | Europe | Detection | Direct | CUA |
| Gomez 2021 | Cost-effectiveness of bedaquiline, pretomanid and linezolid for treatment of extensively drug-resistant tuberculosis in South Africa, Georgia and the Philippines. | BMJ open | Europe | Treatment | Direct | CEA |
| Hickey 2022 | Evaluation of screening strategies for pulmonary tuberculosis among hospitalized patients in a low-burden setting: cost-effectiveness of GeneXpert MTB/RIF compared to smear microscopy. | Infection control and hospital epidemiology | NA | Detection | Direct | CEA |
| Dale 2022 | Modeling the Cost-Effectiveness of Latent Tuberculosis Screening and Treatment Strategies in Recent Migrants to a Low-Incidence Setting. | American journal of epidemiology | Aus | Detection | Direct | CUA |
| Menzies 2022 | The Health and Economic Benefits of Tests That Predict Future Progression to Tuberculosis Disease. | Epidemiology | NA | Detection | Direct | CUA |
| Dodd 2022 | The global impact of household contact management for children on multidrug-resistant and rifampicin-resistant tuberculosis cases, deaths, and health-system costs in 2019: a modelling study | The Lancet Global health | Multiple | Detection | Direct | CEA |
