# Supplementary material for: Inclusion of non-medical interventions in model-based economic evaluations for tuberculosis: A scoping review
Source: PLoS One. 2023 Aug 25;18(8):e0290710. doi: 10.1371/journal.pone.0290710 (PMC10456154; doi:10.1371/journal.pone.0290710)
Supplement: S2 Table — (DOCX) [file pone.0290710.s004.docx]

**Table S2.** Study details of evaluations that focused on non-traditional implementation component of a health intervention (n = 8)

| **Study & country**  **of analysis** | **Intervention Type** | **Type of Evaluation**  **& Time Horizon** | **Perspective** | **Model Type** | **Population** | **Comparator strategies** |
| --- | --- | --- | --- | --- | --- | --- |
| **Studies evaluating a unique 'implementation' component** | | | | | | |
| Perlman 2001^1^  United States | Treatment | CEA  5 years | Healthcare payer | Decision Tree | Participants of a syringe exchange program | (1) Status quo (no intervention) (2) TB screening and directly observed preventative treatment (3) Additional monetary incentive to improve treatment adherence |
| Kominski 2007^2^  United States | Treatment | CUA  Lifetime | Societal | Markov | Adolescents with confirmed LTBI | (1) Usual care (given treatment and education material the clinics routinely provide patients) (2) Peer counsel group (counseled by adolescent who has completed treatment) (3) Contingency contracting (a reward was negotiated between parent and adolescent in exchange for adherence) (4) Combined peer counseling and contingency contracting |
| Pisu 2009^3^  United States | Prevention | CEA  Lifetime | Healthcare payer | Markov | Community dwelling adults representative of contacts investigated for LTBI in Alabama | (1) Traditional concentric circle approach for contact tracing (2) Contact priority model for contact tracing |
| Jit 2011^4^  United Kingdom | Detection | CUA  Lifetime | Healthcare payer | Markov | Hard to reach individuals with active pulmonary TB screened or managed by the Find and Treat service | (1) Status quo (no Find and Treat service)  (2) Having only one component of the program (mobile screening unit or the case management component) (3) Having both parts of the service |
| Wade 2012^5^  Australia | Treatment | CEA  1 year | Healthcare payer | Other | People with TB | (1) Drive around observed therapy (2) Video phone calls for observed therapy |
| Patel 2017^6^  Canada | Treatment | CUA  25 years | Healthcare payer | Microsimulation | Individuals with LTBI who are initiating drug therapy in Canada | (1) Status quo (2) Hypothetical adherence intervention (3) Peer support (4) Two-way text messaging support (5) Enhanced adherence counselling (6) Adherence incentives |
| Salcedo 2021^7^  United States | Treatment | CUA  16 months | Healthcare payer | Markov | People with TB | (1) In person DOT (2) AICure (AI) platform for automated DOT |
| Fekadu 2021^8^  United States | Treatment | CEA  1 year | Healthcare payer | Decision Tree | Adults with TB | (1) DOT versus self-administered therapy before the COVID-19 pandemic (2) Video observed therapy versus self-administered therapy during the COVID-19 pandemic |

Abbreviations: AI: artificial intelligence; CEA: cost-effectiveness analysis; CUA: cost-utility analysis; DOT: directly observed therapy; MDR-TB: multi-drug resistant tuberculosis

**References:**

1. Perlman DC, Gourevitch MN, Trinh C, et al. Cost-effectiveness of tuberculosis screening and observed preventive therapy for active drug injectors at a syringe-exchange program. *Journal of urban health : bulletin of the New York Academy of Medicine*. 2001;78(3):550-567. doi:http://dx.doi.org/10.1093/jurban/78.3.550

2. Kominski GF, Varon SF, Morisky DE, et al. Costs and Cost-Effectiveness of Adolescent Compliance with Treatment for Latent Tuberculosis Infection: Results from a Randomized Trial. *Journal of Adolescent Health*. 2007;40(1):61-68. doi:http://dx.doi.org/10.1016/j.jadohealth.2006.08.012

3. Pisu M, Gerald J, Shamiyeh JE, et al. Targeted tuberculosis contact investigation saves money without sacrificing health. *Journal of public health management and practice : JPHMP*. 2009;15(4):319-327. doi:https://dx.doi.org/10.1097/PHH.0b013e31819c3ef2

4. Jit M, Stagg HR, Aldridge RW, et al. Dedicated outreach service for hard to reach patients with tuberculosis in London: Observational study and economic evaluation. *BMJ (Online)*. 2011;343(7826):d5376. doi:https://dx.doi.org/10.1136/bmj.d5376

5. Wade VA, Karnon J, Eliott JA, et al. Home videophones improve direct observation in tuberculosis treatment: a mixed methods evaluation. *PLoS ONE*. 2012;7(11):e50155. doi:http://dx.doi.org/10.1371/journal.pone.0050155

6. Patel AR, Campbell JR, Sadatsafavi M, et al. Burden of non-adherence to latent tuberculosis infection drug therapy and the potential cost-effectiveness of adherence interventions in Canada: A simulation study. *BMJ Open*. 2017;7(9):e015108. doi:https://dx.doi.org/10.1136/bmjopen-2016-015108

7. Salcedo J, Rosales M, Kim JS, Nuno D, Suen S, Chang AH. Cost-effectiveness of artificial intelligence monitoring for active tuberculosis treatment: A modeling study. *PLoS ONE*. 2021;16(July):1-15. doi:10.1371/journal.pone.0254950

8. Fekadu G, Jiang X, Yao J, You JHS. Cost-effectiveness of video-observed therapy for ambulatory management of active tuberculosis during the COVID-19 pandemic in a high-income country. *International Journal of Infectious Diseases*. 2021;113:271-278. doi:10.1016/j.ijid.2021.10.029
